# MA Cation-Induced Diffusional Growth of Low-Bandgap FA-Cs Perovskites Driven by Natural Gradient Annealing

**DOI:** 10.34133/2021/9765106

**Published:** 2021-08-18

**Authors:** Taiyang Zhang, Yuetian Chen, Miao Kan, Shumao Xu, Yanfeng Miao, Xingtao Wang, Meng Ren, Haoran Chen, Xiaomin Liu, Yixin Zhao

**Affiliations:** ^1^School of Environmental Science and Engineering, Shanghai Jiao Tong University, 800 Dongchuan Rd., Shanghai 200240, China; ^2^Shanghai Institute of Pollution Control and Ecological Security, Shanghai 200240, China

## Abstract

Low-bandgap formamidinium-cesium (FA-Cs) perovskites of FA_1-*x*_Cs*_x_*PbI_3_ (*x* < 0.1) are promising candidates for efficient and robust perovskite solar cells, but their black-phase crystallization is very sensitive to annealing temperature. Unfortunately, the low heat conductivity of the glass substrate builds up a temperature gradient within from bottom to top and makes the initial annealing temperature of the perovskite film lower than the black-phase crystallization point (~150°C). Herein, we take advantage of such temperature gradient for the diffusional growth of high-quality FA-Cs perovskites by introducing a thermally unstable MA^+^ cation, which would firstly form *α*-phase FA-MA-Cs mixed perovskites with low formation energy at the hot bottom of the perovskite films in the early annealing stage. The natural gradient annealing temperature and the thermally unstable MA^+^ cation then lead to the bottom-to-top diffusional growth of highly orientated *α*-phase FA-Cs perovskite, which exhibits 10-fold of enhanced crystallinity and reduced trap density (~3.85 × 10^15^ cm^−3^). Eventually, such FA-Cs perovskite films were fabricated into stable solar cell devices with champion efficiency up to 23.11%, among the highest efficiency of MA-free perovskite solar cells.

## 1. Introduction

Organic-inorganic hybrid metal halide perovskites with superior photovoltaic (PV) performances have drawn enormous research interest within the last few years [1–3]. The power conversion efficiencies (PCEs) of the fabricated perovskite solar cells have dramatically progressed from the unstable 3.8% in 2009 to a certified record of 25.5% in 2020 [4–6]. Beyond the high efficiency, more and more concerns on the long-term stability have risen. Therefore, FA-based perovskite with enhanced thermal stability and a narrower bandgap is a promising candidate to pursue robust and efficient solar cells [7–11]. However, the photoactive black phase (*α* phase) usually requires higher annealing (~170°C) and tends to transform into the inactive nonperovskite yellow phase (*δ* phase). To lower the *α*-phase crystallization temperature and stabilize the black phase, the introduction of smaller cations such as MA^+^ and Cs^+^ or anions like Br^−^ to form mixed cation/anion alloy perovskites has been one of the most popular approaches [8, 12–14]. However, such alloying procedure needs to be carefully tuned as the incorporation of Br^−^ and smaller cations leads to broadening of the perovskites' bandgap and raises concerns on phase segregation [6, 15–20].

The annealing route is another important but often less concerning factor for the fabrication of high-quality FA-based perovskites [21–24]. The regular hotplate thermal annealing process is the most adopted approach for large-scale perovskite deposition. The naturally formed bottom-to-top annealing temperature gradient exists when the glass substrate is placed on the hotplate for thermal annealing due to the low heat conductivity of glass, as shown in Figure S1 [25]. According to previous studies [24], it usually takes several seconds for the entire perovskite precursor film to reach the set temperature. Such unexpected phenomenon may be crucial for a temperature-sensitive process. As the crystallization of FAPbI_3_ is sensitive to the annealing temperature, such temperature gradient might manipulate the FA perovskite of crystallizing into the *δ* phase with low formation energy in the initial annealing stage, leading to an inevitable *δ*-*α*-phase transformation and deterioration of the quality of the final perovskite films [12, 26]. Meanwhile, Pool et al. found that radiative thermal annealing could produce high-quality FAPbI_3_ films because of the faster and more uniform heating flux direction [23]. However, to the best of our knowledge, few research efforts have been invested in utilizing this gradient phenomenon.

In this work, we report the diffusional growth of high-quality low-bandgap FA-Cs perovskites via an intermediate engineering strategy utilizing the annealing temperature gradient. We found that the extra MA^+^ cation in the FA-Cs perovskite precursor could significantly lower the formation energy of *α*-phase perovskite by forming FA-MA-Cs perovskite seeds firstly at the bottom of the perovskite films. Driven by the thermal gradient, such *α*-phase perovskite crystal seeds could diffuse efficiently, thus promoting the bottom-to-top gradient growth of perovskite and suppressing the crystallization of the unwanted *δ* phase. Finally, a high-performance MA-free FA_0.95_Cs_0.05_PbI_3_-based solar cell device with a PCE of over 23% can be obtained. Such value is among the highest efficiency of the MA-free perovskite solar cells. Besides the high efficiency, the stability of the device is also greatly enhanced, which could retain 90% of its initial PCE after 1000 h white LED light soaking.

## 2. Results

**Figure 1:**
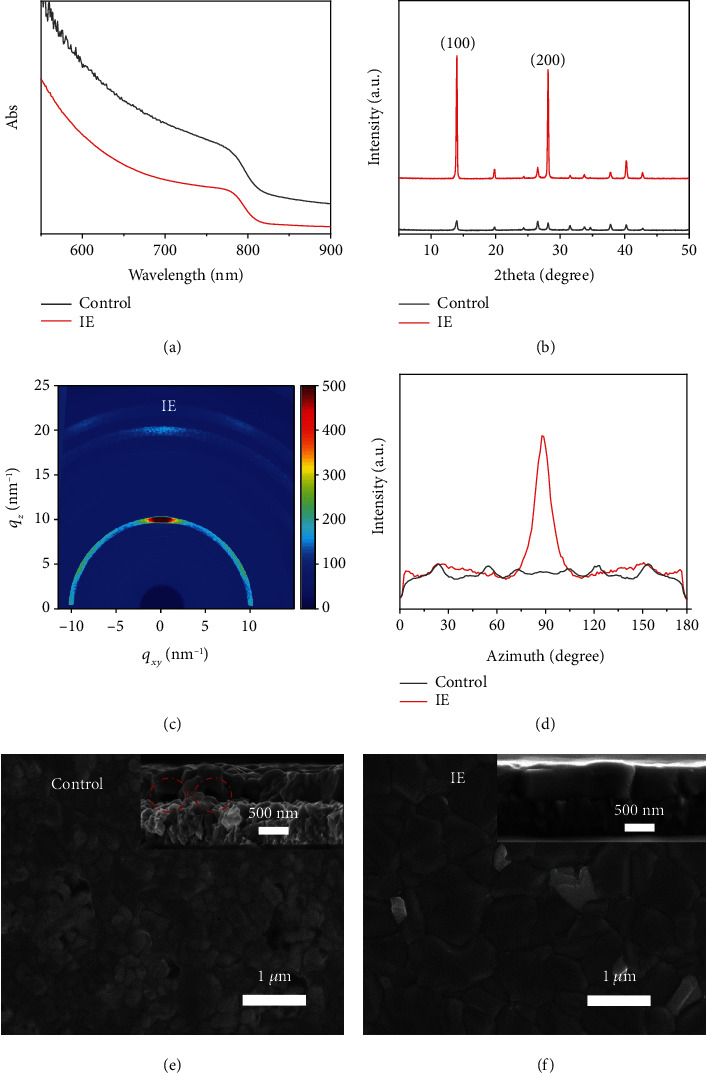
Effect of the intermediate engineering process on the quality of perovskite films. UV-vis spectra (a) and XRD patterns (b) of annealed films with and without IE. GIWAXS pattern of the IE sample (c). Radially integrated intensity plots of the *q* = ~10 nm^–1^ peak from the 2D GIWAXS patterns (d). SEM images of annealed films of the control (e) and IE (f). Corresponding cross-sectional SEM images are shown as inset; the voids between the film and the substrate are marked by red circles.

We chose the FA_0.95_Cs_0.05_PbI_3_ perovskite as the light absorber layer because of its excellent thermal stability and narrow bandgap (~1.53 eV) [27]. In our intermediate engineering process (denoted as IE), different amounts of MAI were added into the FA_0.95_Cs_0.05_PbI_3_ precursors to form a new IE solution (see methods in Supplementary Materials for experimental details). We firstly compared the effect of the new precursor on the final film quality. As shown in Figure 1(a), both the IE sample and the control sample show the same absorption onset at ~813 nm, indicating the successful formation of ~1.53 eV bandgap perovskites. The baseline of the control sample in the UV-vis spectrum is much higher than that of IE, indicating a rougher surface of the control sample. It is worth noting that the two samples had exactly the same absorption onset as the previously reported FA_0.95_Cs_0.05_PbI_3_ perovskite [28]. The photoluminescence (PL) spectra of the IE sample and the control sample (Figure S2) show a peak at the same position (~810 nm) while the PL intensity of the IE sample is much stronger than that of the control sample. Furthermore, NMR and TGA shown in Figure S3 and S4 excluded the existence of MA^+^ residues in the final film, confirming that the IE sample is a pure FA_0.95_Cs_0.05_PbI_3_ perovskite.

Figure 1(b) shows the XRD patterns of the IE and control samples. The IE sample exhibits two strong and sharp peaks at ~13.9 degrees and 28.1 degrees, which could be ascribed to the (100) and (200) plane signals of *α*-phase FA_0.95_Cs_0.05_PbI_3_ perovskite. There are no peaks belonging to the *δ* phase or MAPbI_3_ or impurities in this XRD pattern (Figure S5), indicating a highly crystalline and phase-pure FA_0.95_Cs_0.05_PbI_3_ perovskite film. The peak intensity of the IE perovskite films is almost 10 times stronger than that of the control sample. Synchrotron radiation grazing-incidence wide-angle X-ray scattering (GIWAXS) analysis is then performed to probe the crystal orientation in the perovskite films. As revealed in Figure S6, the control sample shows a weak diffraction ring, indicating the random crystal plane stacking. As for the IE sample (Figure 1(c)), a dark red diffraction mottling could be found at *q*_*z*_ = 10 nm^−1^, clearly revealing the strong (100)-oriented growth of the FA_0.95_Cs_0.05_PbI_3_ films. The azimuth angle plots in Figure 1(d) show the integrated intensity plots of the *q* = ~10 nm^–1^ peak. Compared to the overall low intensity of the control sample, the IE sample demonstrated a sharp peak at ~90 degrees, which further strengthened the preferred (100) orientation of the IE sample [29–31].

SEM images of the IE and control samples are shown in Figures 1(e) and 1(f) and Figure S7. The IE sample exhibits a compact surface and clear grain boundaries with >500 nm grain size, while the control sample shows pinholes with ~200 nm grain size. Moreover, the cross-sectional SEM image of the IE sample (Figure 1(f) inset) shows a quasi-single crystalline feature in the vertical cross-section while the control sample shows polycrystalline grains with lots of voids between the film and the substrate in the vertical direction. The IE films with larger grain size, better substrate contact, and out-of-plane orientation are more favorable for the fabrication of high-efficiency perovskite solar cells, which will be discussed in detail later. The IE sample also demonstrates excellent thermal stability, as shown in Figure S8; the sample shows no visible degradation after aged at a ~100°C hotplate for 10 days. Here, we should note that the amount of excess MA^+^ cations needs to be carefully tuned for a nice film. We found the 0.2MAI sample as the optimal addition amount while too much or less MA^+^ cations would deteriorate the film quality, and the 0.2MAI sample demonstrates the best performance (Figure S9–S11). Therefore, we chose the 0.2MAI recipe for further discussion and optimization.

**Figure 2:**
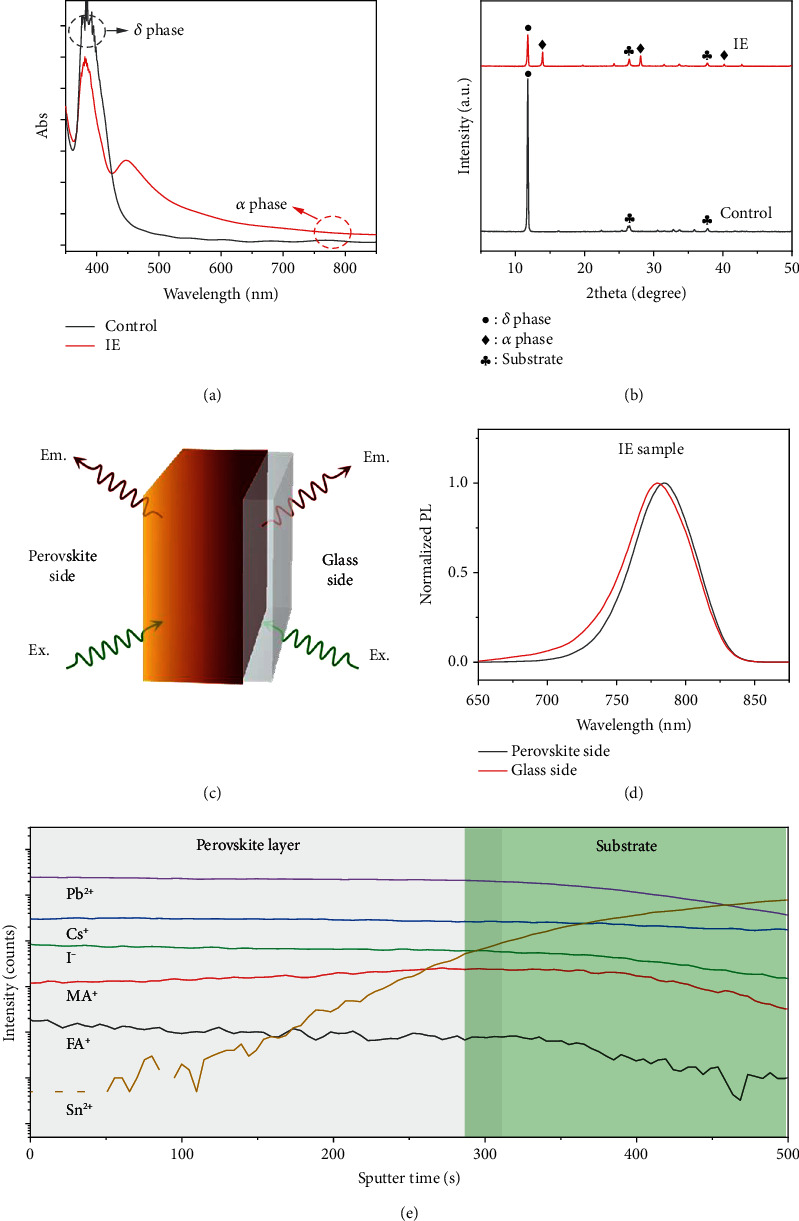
Characterization of the IE and control perovskite precursor films. UV-vis spectra (a) and XRD patterns (b) of the control and IE precursor films. Schematic diagram of the double-side PL test (c). PL spectra (d) measured from both sides of the IE precursor film. ToF-SIMS depth profile of the IE precursor film (e).

The above results confirmed that intermediate engineering could greatly change the film quality; we then explored the role of the extra MA^+^ cation in the crystallization process. Firstly, the as-prepared precursor films, which were annealed at 60°C for 30 s to repel the solvent, were examined. As shown in Figure S12, the IE and control precursor films show a significant difference in color at the initial stage; the IE sample is brown while the control sample is yellowish. UV-vis spectra in Figure 2(a) reveal that only the *δ*-phase absorbance feature (~400 nm) is observed in the control sample, while the IE film has a distinct shoulder peak at ~763 nm and a *δ*-phase-related absorbance peak at ~400 nm. It is highly likely that the phase segregation took place immediately in the IE precursor film with the formation of both *α*-phase perovskite seeds and *δ*-phase perovskites. It is also worth noting that such *α*-phase perovskite seeds have a wider bandgap than the target perovskite, indicating that the precrystallized perovskite seeds could contain some MA^+^ cations. The XRD pattern of the IE precursor film in Figure 2(b) exhibits one strong peak at ~11.7 degrees and another three peaks at ~13.9, 28.1, and 42.2 degrees, which should be assigned to the *δ*-phase and *α*-phase perovskites; no MAPbI_3_ peaks are observed (Figure S13), respectively [32–35]. In contrast, only a very sharp *δ*-phase peak could be observed in the control precursor film. The AFM images in Figure S14 show that the control precursor film has a smaller grain size of 200~500 nm with clear grain boundaries while the grain boundaries of the IE precursor sample are less distinct, confirming that the crystallinity of the IE sample is weaker than that of the control sample. Both the UV-vis absorption and the XRD results suggest that the extra MA^+^ cation in the IE sample may induce the precrystallization of possible *α*-phase FA-Ma-Cs mixed cation perovskite seeds and cause the phase segregation between the *δ* phase and the *α* phase. Additives of MAI and MACl show a similar effect in tuning crystallization dynamics.

Since, in principle, regular UV-vis and XRD analyses yield statistical results, we could not get the spatial distribution of the *α* phase in the films from these characterization techniques alone. We then performed a double-side PL test, which allows us to determine the existence of inhomogeneous growth of perovskite in the films. As illustrated in Figure 2(c), in this test, the PL spectrum collected from the perovskite side is dominated by signals from the top surface of the perovskite film, while the PL spectrum measured from the glass side gives more information on the perovskite adjacent to the glass substrate [36]. As shown in Figure 2(d), the PL spectra collected from two sides of the IE sample show a significant difference: the PL peak collected from the glass side has blue-shifted from the perovskite surface side, indicating that the perovskite seeds in the bottom part have a wider bandgap and could be more MA-rich than the top ones. As for the control sample (Figure S15), the weak PL signals exhibit no variation in peak positions between the two sides. Time-of-flight secondary ion mass spectrometry (ToF-SIMS) depth analysis is performed to further explore the existence of a possible compositional gradient. As shown in Figure 2(e), the signals of Cs^+^, FA^+^, and Pb^2+^ are uniformly distributed across the entire depth of the precursor film, while the signal of MA^+^ is slightly stronger near the bottom than the surface. This disparity in distribution confirms that MA^+^ is richer near the bottom, which is well consistent with the PL results. Here, we could propose a hypothesis that the diffusional growth of *α*-phase perovskite happens across the vertical direction in the IE sample. The precrystallized *α* phase near the bottom is more MA-rich than that near the surface because a higher amount of smaller MA^+^ cations can help to lower the formation energy barrier for the *α* phase [37, 38]. Such diffusional growth then induces the oriented crystallization of high-quality perovskite films, which will be further analyzed later.

**Figure 3:**
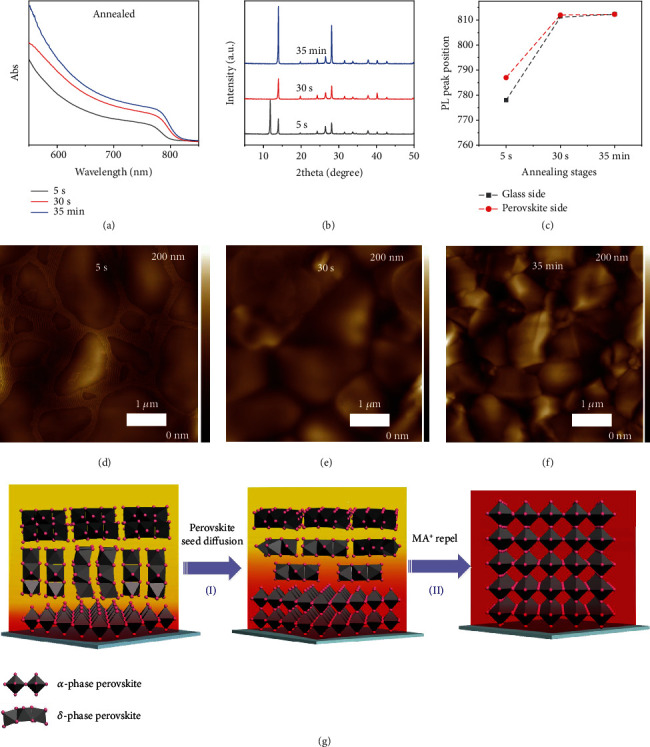
The evolution process of the IE perovskite films within annealing. UV-vis spectra (a), XRD patterns (b), PL peak positions of this double-side test (c), and AFM images (d–f) demonstrating the evolution of the precursor films with different annealing durations. Schematic illustration of the proposed seed diffusion-assisted crystallization of the perovskite films (g).

These material characterization results have proven the coexistence of the *α* phase and the *δ* phase in the IE precursor film, and the *α* phase was gradient-distributed along the vertical direction. As the annealed final film shown in Figure 1(b) is a pure *α* phase, we then investigated the detailed crystallization process of the IE samples to understand the *δ*-*α*-phase transformation. As shown in Figure 3(a), the IE perovskite film's absorbance onset red-shifted from ~801 nm to 813 nm after annealing at 150°C for ~30 s, which is very close to the feature of the final film. Further annealing duration led to the enhancement of absorbance intensity instead of onset shift, indicating a fast-diffusional perovskite phase crystallization through the whole film.

The PL analysis in Figure 3(c) and Figure S16 also shows that after the initial high-temperature annealing, the difference of PL peak positions between the two sides vanished. This observation further confirmed the hypothesis that the MA^+^ cation-contained perovskite seeds would diffuse quickly from bottom to top. The XRD patterns shown in Figure 3(b) also indicate that after 30 s of annealing, the *α*-phase perovskite peaks still existed while the *δ*-phase peak at 11.6 degrees completely disappeared. Moreover, the intensity of perovskite peaks has greatly enhanced after 35 min of annealing. The morphology evolutions characterized by AFM imaging (Figures 3(d)–3(f)) demonstrate similar results. The grain size started to decrease while the grain boundaries became much clearer with annealing, suggesting that the phase and component transformation occurred during the annealing process. The control samples show a quite different evolution process as the UV-vis absorption spectra and XRD patterns revealed in Figure S17 and S18. The typical *α*-phase absorbance starts to rise after 5 min of annealing, and then the absorbance intensity slowly reaches the maximum values after 10 min of annealing. The XRD patterns only show strong *δ*-phase peaks in the initial annealing, and then the *α*-phase peaks start to emerge after 5 min of annealing, but the *δ*-phase peaks still coexist. The *δ*-phase peaks vanished after 10 min of annealing. Such results are well consistent with the UV-vis spectral profiles, further confirming the much slower phase transformation in the control samples.

Based on all the above experimental results, we finally propose a growth mechanism of our IE method. As illustrated in Figure 3(g), the diffusional growth process could be divided into two main steps: the short time step I (~30 s) and the much longer step II (~35 min). In step I, DMSO-containing PbI_2_-DMSO adducts form after the solvent engineering process, which would retard the further crystallization process. As we know, DMSO has a strong capability of coordinating with PbI_2_ and MAI but weakly interacting with FAI, as evidenced by Figure S19 [19, 20, 39]. As the dissociation rate of DMSO is faster via annealing, DMSO molecules close to the hotter bottom are firstly dissociated upon annealing, triggering the crystallization process. The formation of the temperature gradient mainly impacts this step. In the actual experimental settings, the initial film temperature is lower than the value set on the hotplate. This temperature gradient built up inside the perovskite film during thermal annealing leads to the formation of yellow-phase perovskite. Previous works have confirmed that the formation energy plays an important role in the crystallization process of perovskites [38, 40]. The yellow *δ* phase is more favorable for FA_1-*x*_Cs*_x_*PbI_3_ (*x* < 0.1) when the annealing temperature is below the phase transition point (~150°C) [12]. In our intermediate engineering method, the formation energy of black-phase perovskite was greatly reduced by first the formation of FA-MA-Cs perovskite [37, 38]. Upon annealing, the hot bottom helped to dissociate the DMSO molecule and precrystallize the black-phase perovskite seeds in the IE sample. Then, as the annealing proceeds, the crystallization of black-phase perovskite diffused vertically to the top of the film. In such bottom-to-top diffusional growth of FA-Cs perovskite, we could successfully utilize the annealing temperature gradient and the thermally unstable MA-based perovskite seeds. In contrast, if the precursor film is annealed from the top side via hot airflow, the perovskite film shows much-reduced crystallinity than that of the bottom-annealed sample (Figure S20). What is more, the intensity ratio of the peak located at ~14 degrees ((100) plane) and ~20 degrees ((110) plane) was also much smaller than that of the bottom-annealed samples, indicating the less oriented growth of the perovskite film. Such results further confirmed that such temperature gradient is an important factor for the preparation of highly oriented perovskite films. In step II, as the interaction of PbI_2_ and FA^+^ is much stronger than that of MA^+^, the MA^+^ cation was replaced by the FA^+^ and then repelled from the film to further improve the film quality (Figure S21). As evidenced by previous reports [41, 42], such process may last for tens of minutes. Although the duration of step I is relatively short, its importance cannot be ignored. The MA^+^ cation-induced precrystallized perovskite seeds at the bottom in step I could also act as templates for the further growth of perovskite films as they could relieve the strain built during the *δ*-*α*-phase transformation. As we know, the *δ* phase consists of face-sharing PbI_6_ octahedra while the PbI_6_ octahedron in *α*-phase perovskite is corner-sharing. The face-sharing mode is more compact than the corner-sharing mode; hence, an expansion of the PbI_6_ octahedron would take place in this phase transformation process [26]. Such feature is somehow similar to the often-considered difficult “two-step” method, which involves the transformation from highly crystalline PbI_2_ to MAPbI_3_ [43–47]. For the control sample, the highly crystalline *δ* phase is so hard to corrode, leading to the smaller crystal size and formation of pinholes in the films [26]. This difficult *δ*-*α*-phase transformation also caused more critical lattice tension/compression within the perovskite film, which could lead to more serious strain [48]. Such strains could induce more ion migration and generate more defect sites, which not only deteriorated the device performance but also reduced the stability of perovskite films [49, 50]. On the contrary, the crystallinity of the *δ* phase in the IE sample was greatly reduced by the perovskite seeds. Thus, the expansion of the PbI_6_ octahedron was promoted and the lattice stains were relieved, which finally leads to the growth of highly crystalline, well-oriented, pinhole-free, and robust perovskite films.

**Figure 4:**
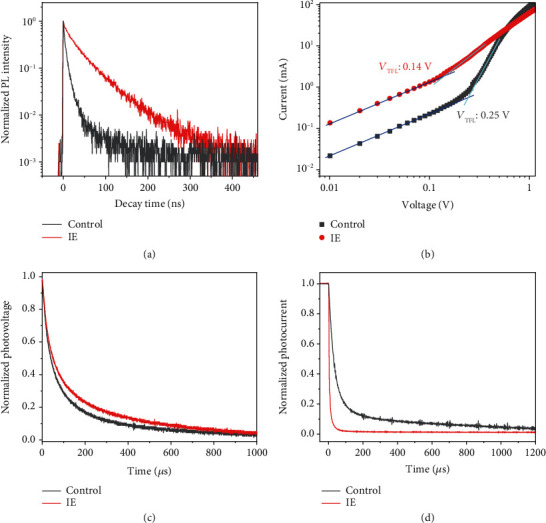
Charge transfer properties of the IE and control samples. TRPL curves (a) of the IE films and the control films. Dark *I*-*V* curves (b) of the IE- and control-based electron-only devices. TPV (c) and TPC (d) curves of the IE and control devices.

The impact of our IE method on the charge transfer dynamics was further investigated. Time-resolved photoluminescence (TRPL) results are shown in Figure 4(a), and the PL lifetime could be obtained by biexponential fitting using the following equation:

(1) $$\begin{array}{c} Y=\text{A}1\exp\left(-t/\tau_{1}\right)+\text{A}2\exp\left(-t/\tau_{2}\right). \end{array}$$

The IE sample shows a much longer average PL lifetime (32.81 ns) than the control sample (5.15 ns), indicating that the trap state-assisted nonradiative recombination is greatly suppressed in the IE sample [51]. The electron-only devices using the FTO/TiO_2_-SnO_2_/perovskite/PCBM/Ag structure were also fabricated to evaluate the trap density of the IE and control samples. As shown in Figure 4(b), the linear section (blue line) at low bias voltage indicates the ohmic-type response, the trap filling region is marked by the light cyan line, and the trap filled limit voltage (*V*_TFL_) lies in the kink point of the two regions. The trap density (*N*_t_) of perovskites can be calculated by the following equation:

(2) $$\begin{array}{c} N_{\text{t}}=\frac{2\varepsilon_{0}\varepsilon_{\text{r}}V_{\text{TFL}}}{eL^{2}}, \end{array}$$

where the *ε*_0_ and *ε*_r_ represent the vacuum permittivity and the relative dielectric constant of perovskites (*ε* = 62.23 for FA_0.95_Cs_0.05_PbI_3_) [28], *e* is the electron charge, and *L* denotes the thickness of the films. The trap density for the control sample is ~6.88 × 10^15^ cm^−3^ while the IE sample shows a much-reduced value of ~3.85 × 10^15^ cm^−3^. Such remarkable lower trap density should be benefited from the larger grain size and the well-oriented growth of IE samples. The transient photovoltage (TPV) decay result in Figure 4(c) shows that the IE sample has a longer charge-carrier lifetime than the control sample, indicating the lower undesired carrier's recombination rate of the IE sample, which is consistent with the TRPL result. Moreover, as shown in Figure 4(d), the shorter lifetime of the IE sample in the transient photocurrent (TPC) decay curves also confirmed that the IE sample had a much faster photocarrier transit rate than the control sample. All the results further strengthen the fact that the high-quality IE film can reduce the defect density and facilitate the carrier transfer and extraction [49, 52].

**Figure 5:**
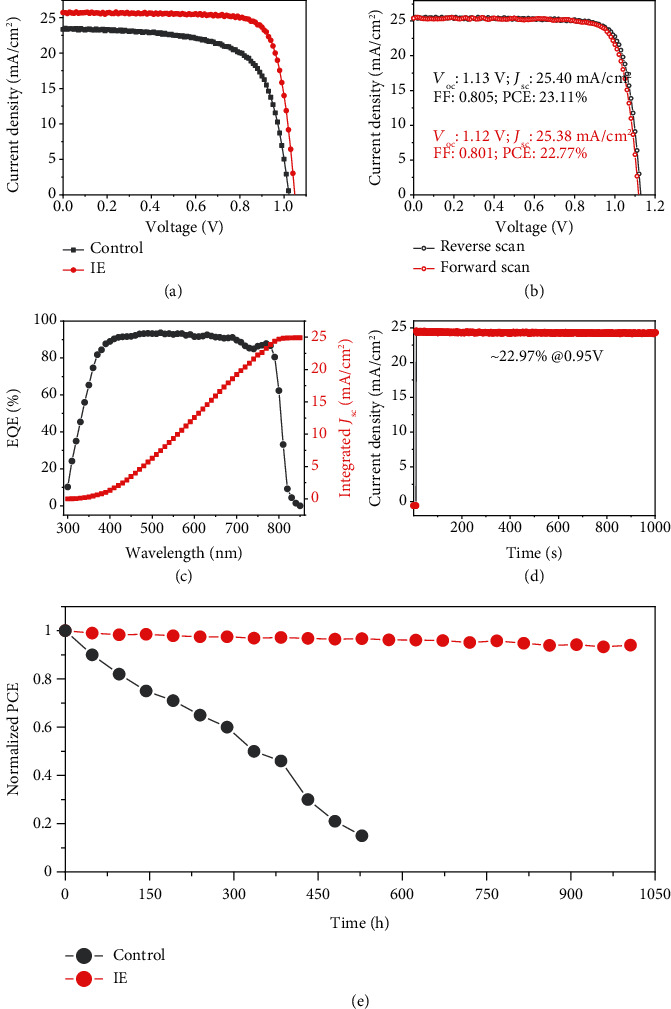
Performance of the fabricated FA_0.95_Cs_0.05_PbI_3_-based solar cell devices. Typical *J*-*V* curves (a) of the IE and control perovskite devices. *J*-*V* curve (b), EQE curve (c), and stable output curve (d) of the champion IE device. Stability test (e) of the IE and control devices. The devices were irradiated under a white LED light source (100 mW/cm^2^) in an N_2_ glovebox.

The high-quality perovskite films were then fabricated into perovskite solar cell devices using the FTO/TiO_2_-SnO_2_/perovskite/Spiro-OMeTAD/Ag planar architecture. As shown in Figure 5(a) and Table S1, the IE devices show much-enhanced performance with a PCE increasing from ~15% to ~21% and better reproducibility. A champion IE-based device reached a PCE of 23.11% with an open-circuit voltage (*V*_oc_) of 1.13 V, a fill factor (FF) of 0.805, and a short-circuit photocurrent density (*J*_sc_) of 25.40 mA/cm^2^ (Figure 5(b)), which is much better than the control sample. The *J*_sc_ value (25.40 mA/cm^2^) obtained from the *J*-*V* curve is well consistent with the integrated value (24.9 mA/cm^2^) from the external quantum efficiency (EQE) curve in Figure 5(c). Negligible hysteresis was also observed, and a stable output efficiency of 22.97% was obtained (Figure 5(d)). Beyond the high efficiency, the stability of devices is also greatly enhanced. The IE device could retain 90% of its initial performance after 1000 h white LED light soaking, while the performance of the control device deteriorated dramatically (Figure 5(e)). The much-enhanced stability of the IE device could be attributed to its better crystallinity and less trap density, relieving the strains that inhibited the defect-triggered degradation.

## 3. Discussion

In summary, we developed an intermediate engineering method for the growth of highly crystalline phase-pure *α*-FA_0.95_Cs_0.05_PbI_3_ perovskites. Further investigations revealed that the precrystallization of *α*-phase perovskite seeds could be triggered by the addition of excess MA^+^ cations, which lowered the formation energy of the *α*-phase perovskite and took advantage of the temperature gradient effect during the initial annealing process. The bottom-to-top growth of perovskite was then promoted, and the strain within the growth of perovskite was relieved. The obtained films have crystallites well oriented vertically. The film quality was greatly improved with enhanced crystallinity and reduced trap density, thus suppressing the nonradiative recombination and promoting the carrier transfer and extraction. Finally, the high-quality *α*-FA_0.95_Cs_0.05_PbI_3_ perovskite-based solar cells exhibited much-enhanced PV performance, and the champion device reached an efficiency of over 23% with negligible *J*-*V* hysteresis. Our intermediate engineering approach highlights the importance of the previously neglected annealing temperature gradient within the annealing process. Careful tuning of this gradient would be a promising tactic for the deposition of low-bandgap FA-based perovskites and other hybrid halide perovskites for higher device performances and scalable film deposition.

## Data Availability

The data that support the plots within this paper and other findings of this study are available from the corresponding author upon reasonable request.

## Supplementary Materials

Supplementary Materials Materials and methods. Fig. S1: (a) the temperature profile evolution measured on the top of substrates during the initial annealing process; (b) the temperature was measured using a precise thermometer with a thermal coupler placed on the top of substrates. Fig. S2: PL spectra of perovskite films prepared from the IE and control precursors. Fig. S3: ^1^H NMR spectra of FA_0.95_Cs_0.05_PbI_3_ perovskite films prepared from our IE method. Fig. S4: TGA and the first derivative of TGA curves for FAPbI_3_ at a heating rate of 10°C min^−1^ in N_2_. Fig. S5: enlarged XRD pattern of Figure 1(b). Fig. S6: GIWAXS pattern of the annealed control sample. Fig. S7: cross-sectional SEM images of the perovskite films prepared from the IE and control precursor solutions. Fig. S8: UV-vis spectra of IE perovskite films before and after aged on a 100°C hotplate for 10 days in an N_2_ glovebox. Fig. S9: comparison of SEM images of the annealed perovskite films with different MAI contents; the scale bar is 1 *μ*m. Fig. S10: comparison of UV-vis spectra of annealed perovskite films with different MAI contents. Fig. S11: *J*-*V* curves of perovskite solar cell devices fabricated by precursors with different MAI contents. Fig. S12: photos of perovskite films using the control (a) and IE precursors (b) at the initial (top) and final (bottom) annealing stages. Fig. S13: enlarged XRD pattern of Figure 2(b). Fig. S14: AFM images of the IE (a) and control (b) precursor films. Fig. S15: the PL spectra of the control precursor film collected from the perovskite side and the glass side. Fig. S16: the PL spectra of the annealed IE film collected from the perovskite side and the glass side. Fig. S17: the UV-vis spectral evolution of the control films during the annealing process. Fig. S18: XRD pattern evolution of the control precursor films with different annealing durations. Fig. S19: Fourier transform infrared spectroscopy (FT-IR) spectra of the IE precursor film after antisolvent dipping. Fig. S20: XRD patterns of the IE precursor films with different annealing procedures. Fig. S21: ToF-SIMS of the annealed control and IE samples. Table S1: summary on device performance parameters of FA_0.95_Cs_0.05_PbI_3_ (both the IE and control types)-based perovskite solar cells from 32 devices.

## References

[1] Park N.-G., Grätzel M., Miyasaka T., Zhu K., Emery K. (2016). Towards stable and commercially available perovskite solar cells. *Nature Energy*.

[2] Correa-Baena J.-P., Saliba M., Buonassisi T. (2017). Promises and challenges of perovskite solar cells. *Science*.

[3] Jena A. K., Kulkarni A., Miyasaka T. (2019). Halide perovskite photovoltaics: background, status, and future prospects. *Chemical Reviews*.

[4] Kojima A., Teshima K., Shirai Y., Miyasaka T. (2009). Organometal halide perovskites as visible-light sensitizers for photovoltaic cells. *Journal of the American Chemical Society*.

[5] Kim H.-S., Lee C. R., Im J. H. (2012). Lead iodide perovskite sensitized all-solid-state submicron thin film mesoscopic solar cell with efficiency exceeding 9%. *Scientific Reports*.

[6] Jiang Q., Zhao Y., Zhang X. (2019). Surface passivation of perovskite film for efficient solar cells. *Nature Photonics*.

[7] Pang S., Hu H., Zhang J. (2014). NH2CH═NH2PbI3: an alternative organolead iodide perovskite sensitizer for mesoscopic solar cells. *Chemistry of Materials*.

[8] Jeon N. J., Noh J. H., Yang W. S. (2015). Compositional engineering of perovskite materials for high-performance solar cells. *Nature*.

[9] Amat A., Mosconi E., Ronca E. (2014). Cation-induced band-gap tuning in organohalide perovskites: interplay of spin–orbit coupling and octahedra tilting. *Nano Letters*.

[10] Quarti C., Mosconi E., Ball J. M. (2016). Structural and optical properties of methylammonium lead iodide across the tetragonal to cubic phase transition: implications for perovskite solar cells. *Energy & Environmental Science*.

[11] Juarez-Perez E. J., Hawash Z., Raga S. R., Ono L. K., Qi Y. (2016). Thermal degradation of CH3NH3PbI3 perovskite into NH3 and CH3I gases observed by coupled thermogravimetry-mass spectrometry analysis. *Energy & Environmental Science*.

[12] Li Z., Yang M., Park J. S., Wei S. H., Berry J. J., Zhu K. (2016). Stabilizing perovskite structures by tuning tolerance factor: formation of formamidinium and cesium lead iodide solid-state alloys. *Chemistry of Materials*.

[13] McMeekin D. P., Sadoughi G., Rehman W. (2016). A mixed-cation lead mixed-halide perovskite absorber for tandem solar cells. *Science*.

[14] Xie L., Lin K., Lu J. (2019). Efficient and stable low-bandgap perovskite solar cells enabled by a CsPbBr3-cluster assisted bottom-up crystallization approach. *Journal of the American Chemical Society*.

[15] Saliba M., Matsui T., Seo J. Y. (2016). Cesium-containing triple cation perovskite solar cells: improved stability, reproducibility and high efficiency. *Energy & Environmental Science*.

[16] Hoke E. T., Slotcavage D. J., Dohner E. R., Bowring A. R., Karunadasa H. I., McGehee M. D. (2015). Reversible photo-induced trap formation in mixed-halide hybrid perovskites for photovoltaics. *Chemical Science*.

[17] Min H., Kim M., Lee S. U. (2019). Efficient, stable solar cells by using inherent bandgap of *α*-phase formamidinium lead iodide. *Science*.

[18] Turren-Cruz S.-H., Hagfeldt A., Saliba M. (2018). Methylammonium-free, high-performance and stable perovskite solar cells on a planar architecture. *Science*.

[19] Lee J.-W., Kim H.-S., Park N.-G. (2016). Lewis acid–base adduct approach for high efficiency perovskite solar cells. *Accounts of Chemical Research*.

[20] Lee J.-W., Dai Z., Lee C. (2018). Tuning molecular interactions for highly reproducible and efficient formamidinium perovskite solar cells via adduct approach. *Journal of the American Chemical Society*.

[21] You P., Li G., Tang G., Cao J., Yan F. (2020). Ultrafast laser-annealing of perovskite films for efficient perovskite solar cells. *Energy & Environmental Science*.

[22] Troughton J., Carnie M. J., Davies M. L. (2016). Photonic flash-annealing of lead halide perovskite solar cells in 1 ms. *Journal of Materials Chemistry A*.

[23] Pool V. L., Dou B., van Campen D. G. (2017). Thermal engineering of FAPbI_3_ perovskite material via radiative thermal annealing and *in situ* XRD. *Nature Communications*.

[24] Li G., Zhang T., Xu F., Zhao Y. (2017). A facile deposition of large grain and phase pure *α*-FAPbI_3_ for perovskite solar cells via a flash crystallization. *Materials Today Energy*.

[25] Rolston N., Bennett-Kennett R., Schelhas L. T. (2020). Comment on “Light-induced lattice expansion leads to high-efficiency perovskite solar cells”. *Science*.

[26] Liu T., Zong Y., Zhou Y. (2017). High-performance formamidinium-based perovskite solar cells via microstructure-mediated *δ*-to-*α*-phase transformation. *Chemistry of Materials*.

[27] Lee J.-W., Kim D. H., Kim H. S., Seo S. W., Cho S. M., Park N. G. (2015). Formamidinium and cesium hybridization for photo- and moisture-stable perovskite solar cell. *Advanced Energy Materials*.

[28] Yang D., Yang R., Wang K. (2018). High efficiency planar-type perovskite solar cells with negligible hysteresis using EDTA-complexed SnO_2_. *Nature Communications*.

[29] Kim D. H., Park J., Li Z. (2017). 300% enhancement of carrier mobility in uniaxial-oriented perovskite films formed by topotactic-oriented attachment. *Advanced Materials*.

[30] Xie F., Chen C. C., Wu Y. (2017). Vertical recrystallization for highly efficient and stable formamidinium-based inverted-structure perovskite solar cells. *Energy & Environmental Science*.

[31] Zheng G., Zhu C., Ma J. (2018). Manipulation of facet orientation in hybrid perovskite polycrystalline films by cation cascade. *Nature Communications*.

[32] Eperon G. E., Stranks S. D., Menelaou C., Johnston M. B., Herz L. M., Snaith H. J. (2014). Formamidinium lead trihalide: a broadly tunable perovskite for efficient planar heterojunction solar cells. *Energy & Environmental Science*.

[33] Qifeng H., Bae S.-H., Sun P. (2016). Single crystal formamidinium lead iodide (FAPbI 3): insight into the structural, optical, and electrical properties. *Advanced Materials*.

[34] Xie L.-Q., Chen L., Nan Z. A. (2017). Understanding the cubic phase stabilization and crystallization kinetics in mixed cations and halides perovskite single crystals. *Journal of the American Chemical Society*.

[35] Chen L., Tan Y. Y., Chen Z. X. (2019). Toward long-term stability: single-crystal alloys of cesium-containing mixed cation and mixed halide perovskite. *Journal of the American Chemical Society*.

[36] Lin Y., Fang Y., Zhao J. (2019). Unveiling the operation mechanism of layered perovskite solar cells. *Nature Communications*.

[37] Yi C., Luo J., Meloni S. (2016). Entropic stabilization of mixed A-cation ABX3metal halide perovskites for high performance perovskite solar cells. *Energy & Environmental Science*.

[38] Kim M., Kim G. H., Lee T. K. (2019). Methylammonium chloride induces intermediate phase stabilization for efficient perovskite solar cells. *Joule*.

[39] Jeon N. J., Noh J. H., Kim Y. C., Yang W. S., Ryu S., Seok S. I. (2014). Solvent engineering for high-performance inorganic-organic hybrid perovskite solar cells. *Nature Materials*.

[40] Shao S., Liu J., Portale G. (2018). Highly reproducible Sn-based hybrid perovskite solar cells with 9% efficiency. *Advanced Energy Materials*.

[41] Ma L., Guo D., Li M. (2019). Temperature-dependent thermal decomposition pathway of organic–inorganic halide perovskite materials. *Chemistry of Materials*.

[42] Li C., Zhou Y., Wang L. (2017). Methylammonium-mediated evolution of mixed-organic-cation perovskite thin films: a dynamic composition-tuning process. *Angewandte Chemie, International Edition*.

[43] Burschka J., Pellet N., Moon S. J. (2013). Sequential deposition as a route to high-performance perovskite-sensitized solar cells. *Nature*.

[44] Wu Y., Islam A., Yang X. (2014). Retarding the crystallization of PbI2for highly reproducible planar-structured perovskite solar cells via sequential deposition. *Energy & Environmental Science*.

[45] Yang W. S., Noh J. H., Jeon N. J. (2015). High-performance photovoltaic perovskite layers fabricated through intramolecular exchange. *Science*.

[46] Zhang T., Yang M., Zhao Y., Zhu K. (2015). Controllable sequential deposition of planar CH3NH3PbI3 perovskite films via adjustable volume expansion. *Nano Letters*.

[47] Zhao Y., Tan H., Yuan H. (2018). Perovskite seeding growth of formamidinium-lead-iodide-based perovskites for efficient and stable solar cells. *Nature Communications*.

[48] Chen B., Yu Z., Liu K. (2019). Grain engineering for perovskite/silicon monolithic tandem solar cells with efficiency of 25.4%. *Joule*.

[49] Zhu C., Niu X., Fu Y. (2019). Strain engineering in perovskite solar cells and its impacts on carrier dynamics. *Nature Communications*.

[50] Zhao J., Deng Y., Wei H. (2017). Strained hybrid perovskite thin films and their impact on the intrinsic stability of perovskite solar cells. *Science Advances*.

[51] Wei M., Xiao K., Walters G. (2020). Combining efficiency and stability in mixed tin–lead perovskite solar cells by capping grains with an ultrathin 2D layer. *Advanced Materials*.

[52] Meng L., Sun C., Wang R. (2018). Tailored phase conversion under conjugated polymer enables thermally stable perovskite solar cells with efficiency exceeding 21%. *Journal of the American Chemical Society*.

