# Supplementary material for: MA Cation-Induced Diffusional Growth of Low-Bandgap FA-Cs Perovskites Driven by Natural Gradient Annealing
Source: Research (Wash D C). 2021 Aug 18;2021:9765106. doi: 10.34133/2021/9765106 (PMC8391048; doi:10.34133/2021/9765106)
Supplement: Supplementary Materials — Materials and methods. Fig. S1: (a) the temperature profile evolution measured on the top of substrates during the initial annealing process; (b) the temperature was measured using a precise thermometer with a thermal coupler placed on the top of substrates. Fig. S2: PL spectra of perovskite films prepared from the IE and control precursors. Fig. S3: 1H NMR spectra of FA0.95Cs0.05PbI3 perovskite films prepared from our IE method. Fig. S4: TGA and the first derivative of TGA curves for FAPbI3 at a heating rate of 10°C min−1 in N2. Fig. S5: enlarged XRD pattern of Figure 1(b). Fig. S6: GIWAXS pattern of the annealed control sample. Fig. S7: cross-sectional SEM images of the perovskite films prepared from the IE and control precursor solutions. Fig. S8: UV-vis spectra of IE perovskite films before and after aged on a 100°C hotplate for 10 days in an N2 glovebox. Fig. S9: comparison of SEM images of the annealed perovskite films with different MAI contents; the scale bar is 1 μm. Fig. S10: comparison of UV-vis spectra of annealed perovskite films with different MAI contents. Fig. S11: J-V curves of perovskite solar cell devices fabricated by precursors with different MAI contents. Fig. S12: photos of perovskite films using the control (a) and IE precursors (b) at the initial (top) and final (bottom) annealing stages. Fig. S13: enlarged XRD pattern of Figure 2(b). Fig. S14: AFM images of the IE (a) and control (b) precursor films. Fig. S15: the PL spectra of the control precursor film collected from the perovskite side and the glass side. Fig. S16: the PL spectra of the annealed IE film collected from the perovskite side and the glass side. Fig. S17: the UV-vis spectral evolution of the control films during the annealing process. Fig. S18: XRD pattern evolution of the control precursor films with different annealing durations. Fig. S19: Fourier transform infrared spectroscopy (FT-IR) spectra of the IE precursor film after antisolvent dipping. Fig. S20: XRD patterns of th [file 9765106.f1.docx]

Supporting Information

Title

MA Cation Induced Diffusional Growth of Low Bandgap FA-Cs Perovskites Driven by Natural Gradient Annealing

Diffusional Growth of Low Bandgap FA-Cs Perovskites

**Authors**

Taiyang Zhang^1^, Yuetian Chen^1^, Miao Kan^1^, Shumao Xu^1^, Yanfeng Miao^1^, Xingtao Wang^1^, Meng Ren^1^, Haoran Chen^1^, Xiaomin Liu^1^ and Yixin Zhao^1,2^*

**Affiliations**

- 1, School of Environmental Science and Engineering, Shanghai Jiao Tong University, 800 Dongchuan Rd., Shanghai 200240, China..
- 2, Shanghai Institute of Pollution Control and Ecological Security, Shanghai 200240, China.

Correspondence should be addressed to Yixin Zhao; [yixin.zhao@sjtu.edu.cn](mailto:yixin.zhao@sjtu.edu.cn)

**Materials and methods**

**Materials.** PbI_2_ (99.9985%), CsI (99.9985%), Octylammonium bromide and SnO_2_ (15% in H_2_O colloidal dispersion) were purchased from Alfa Aesar. FAI and MAI were purchased from Shanghai MaterWin New Materials Co. Ltd. All the other materials were purchased from Sigma-Aldrich and used as received without any purification.

**Device fabrication.** Patterned fluorine-doped tin oxide (FTO, TEC 7) glasses with 2.5 mm thickness were cleaned by soaking in 2% NaOH ethanol solution for 12 h, rinsing with DI water, and drying with N_2_ flow. The cleaned substrates were then deposited with a ~20 nm thick compact TiO_2_ layer by spray pyrolysis of 0.2 M Ti (IV) bis (ethyl acetoacetate)-diisopropoxide in 1-butanol solution at 450 ℃ followed by one-hour annealing. Subsequently, SnO_2_ layer was spin coated using 0.75% SnO_2_ solution at 4000 RPM for 30 s, annealed at 150 °C in open air and then treated with UV-O_3_ for 20 min. The IE perovskite precursor solution was made by dissolving 1 mmol PbI_2_, 0.95 mmol FAI and 0.05 mmol CsI powder into 0.689 mL DMF/DMSO mixture solution (v:v=4:1) to form a 1.45 M solution, 0.2 mmol MAI powder was also added to the solution. MACl additives show similar effect on device performance. The control precursor solution was prepared with the same procedure and chemicals but without the extra MAI powders. Perovskite films were deposited by spin coating the precursor solution at 5000 RPM for 20 s. At 10s before the end time, 400 µL CB was dropped on the substrate. The films were then annealed at 150 °C for 35 min in atmosphere with ~30% R.H. The perovskite films were then treated by spin-coating 8 mg/mL octylammonium bromide dichloromethane solution at 5000 RPM for 30s and then annealing at 100 °C for 5 min. The annealed films were spin coated with a layer of hole transport material (HTM) solution consisting of 0.1 M spiro-MeOTAD, 0.035 M bis (trifluoromethane) sulfonimide lithium salt (Li-TFSi), and 0.12 M 4-tert-butylpyridine (TBP) in chlorobenzene/acetonitrile (10:1, v/v) at 4000 RPM for 20 s and then aged overnight before evaporation. All processes were operated in a dry box with less than 20% humidity. Finally, ~100 nm thick Ag layer was thermal evaporated as the back contact.

**Characterization.** The crystal structures of the perovskite films before and after annealing were characterized on X-ray diffraction diffractometer (Shimadzu XRD-6100 diffractometer with Cu Kα radiation). The UV-vis spectra of the films were measured on a Cary-60 UV-vis spectrophotometer. The morphologies of the prepared films were characterized by a JSM-7800F Prime scanning electron microscope (SEM) and a Bruker Multi Mode Nanoscope IIIA atomic force microscope (AFM). The steady and time resolved photoluminescence (PL) spectra were collected from a FLS 1000 photoluminescence spectrometer using bare glasses as substrate. The Nuclear magnetic resonance (NMR) tests were performed on a Bruker AVANCE III 600MHz NMR Instrument using DMSO-d6 as solvent. The GIWAXS tests were performed at BL14B1 line station of Shanghai Synchrotron Radiation facility, the beam energy was 10 keV and the λ was 0.12398 nm. Time-of-flight secondary ion mass spectrometry (ToF-SIMS) depth analysis was performed on a TOF-SIMS 5-100 spectrometer (ION-TOF GmbH) using GCIB as sputter source.The TPC and TPV analysis were collected by Zahner PP211 and Zahner Zennium using a microsecond pulse of a white light incident on solar cells under short circuit condition and open circuit condition. The TGA analysis was carried out on a PerkinElmer TGA-8000 equipment at 10°C/min under N_2_. The photocurrent density–voltage (J–V) curves of perovskite solar cells was measured by a Keithley 2401 source meter under simulated AM 1.5G illumination with a scan rate of 0.05 V/S (100 mW/cm^2^; Enlitech SS-F5-3A Class AAA Solar Simulator, the light intensity was calibrated by a standard Si cell) equipped with a non-reflective metal mask with an aperture area of 0.12 cm^2^. The EQE was measured on a QE-3011 system from Enlitech. All the J-V and EQE tests were processed in atmosphere with R.H 30~45%.


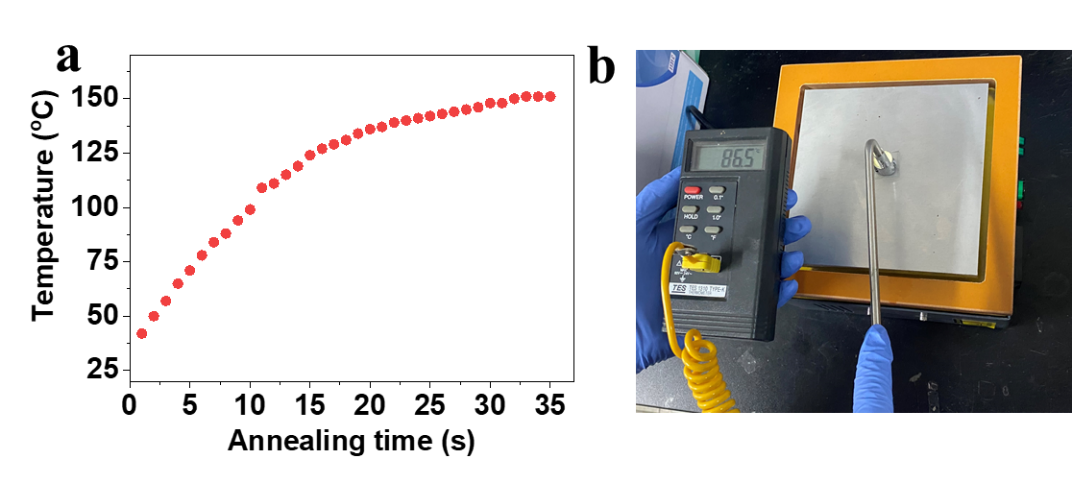


**Figure S1**. (a) The temperature profiles evolution measured on the top of substrates during the initial annealing process. The thickness of the FTO substrate was 2.5 mm. (b)The temperature was measured using a precise thermometer with a thermal coupler placed on the top of substrates.

**Figure S2.** PL spectra of perovskite films prepared from the IE and control precursors.


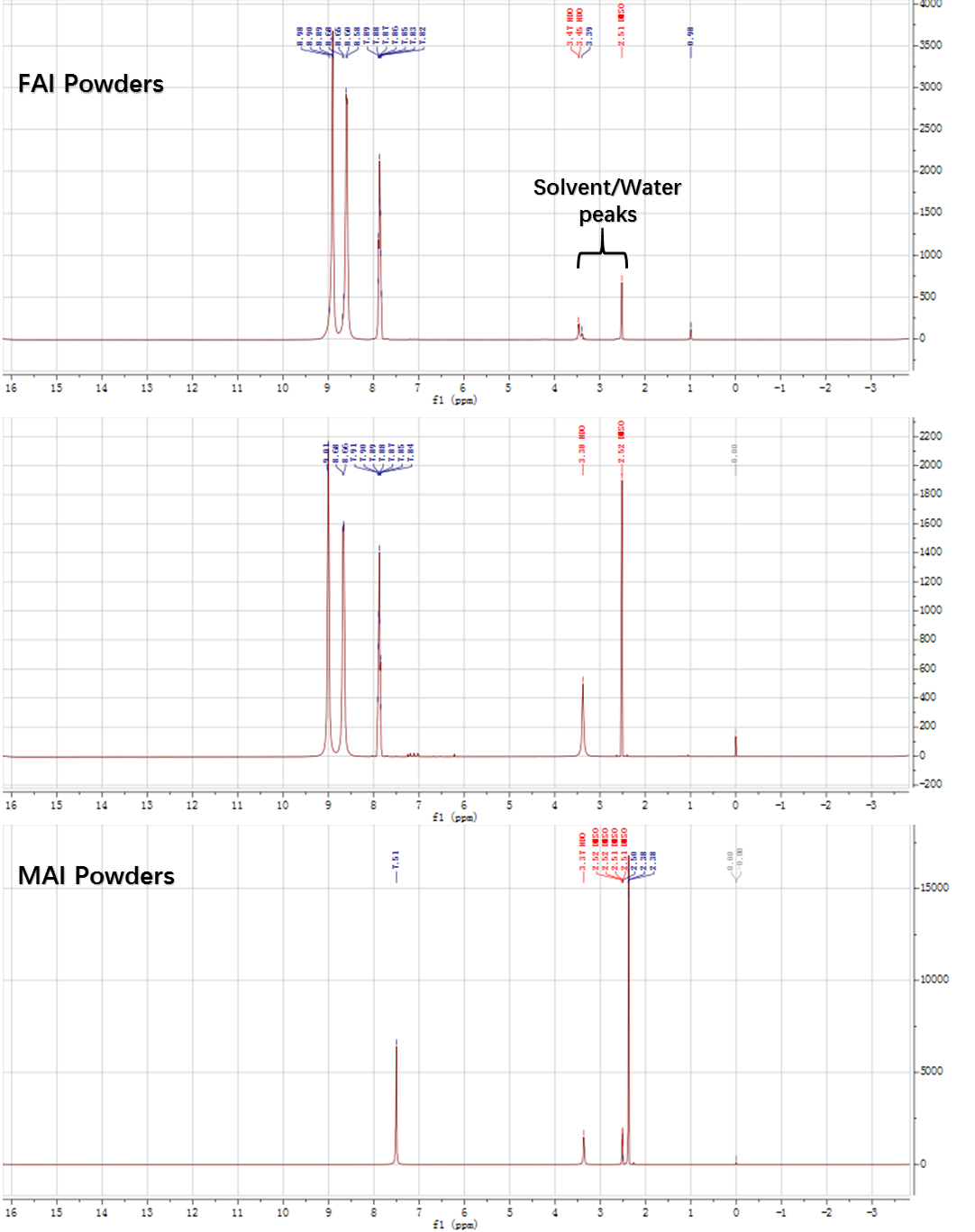


**Figure S3.** ^1^H NMR spectra of FA_0.95_Cs_0.05_PbI_3_ powders scraped from the perovskite films prepared from our IE method. Only peaks from FA group, DMSO-d6 solvent, and H_2_O can be found.

**Figure S4**. TGA and the first derivative of TGA curves for FAPbI_3_ at a heating rate of 10 °C min^−1^ in N_2_. The IE samples experienced ~24.9% weight loss at around 410 °C, which correlates well with the calculated weight fraction of the organic component of FAI in FA_0.95_Cs_0.05_PbI_3_ (25.2%), indicated only FAI organic component in the annealed samples. The 1^st^ derivative curve also show a weight loss peak at ~371 °C, which is also well consisted with previous reported FA-based perovskite (Ref. 41).

**Figure S5**. Enlarged XRD pattern of Figure 1b, no peaks of MAPbI_3_ could be observed.


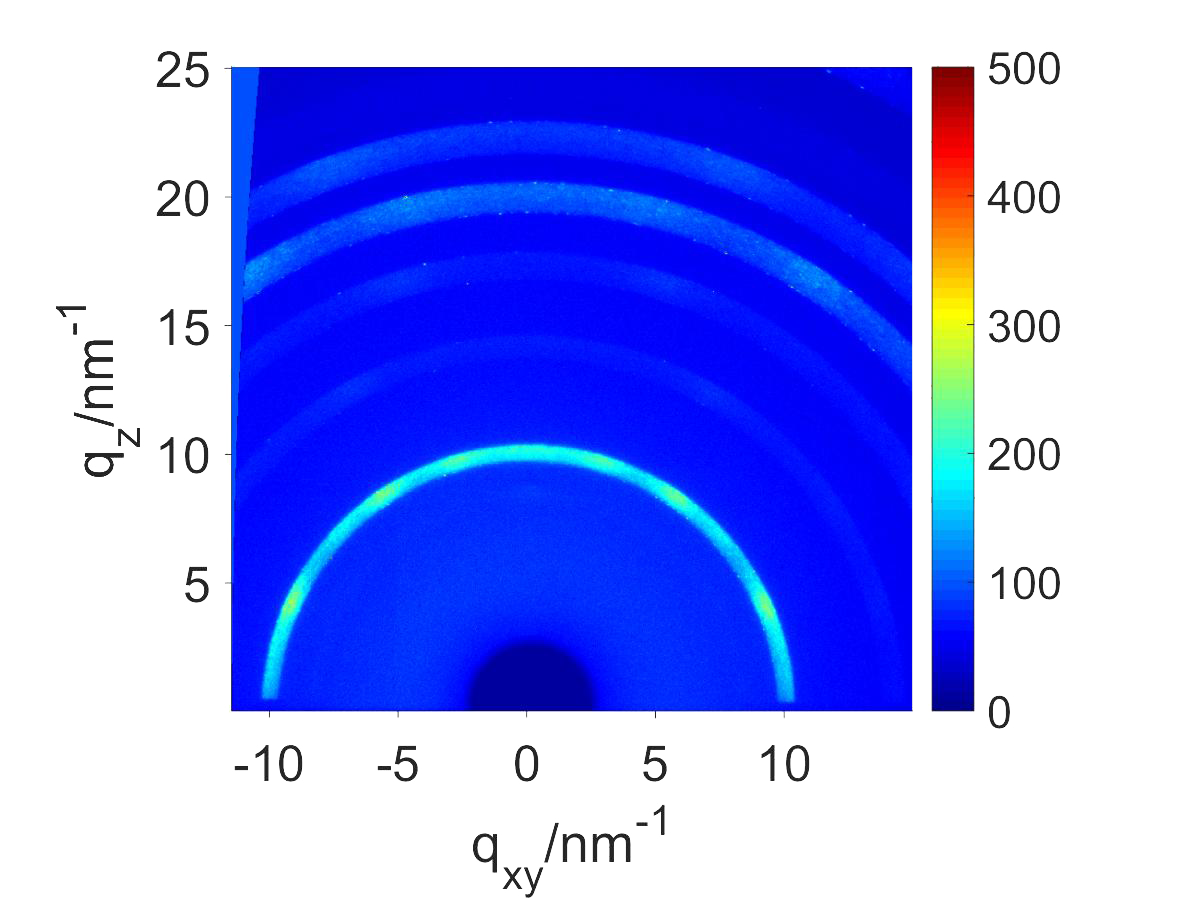


**Figure S6.** GIWAXS pattern of the annealed control sample.


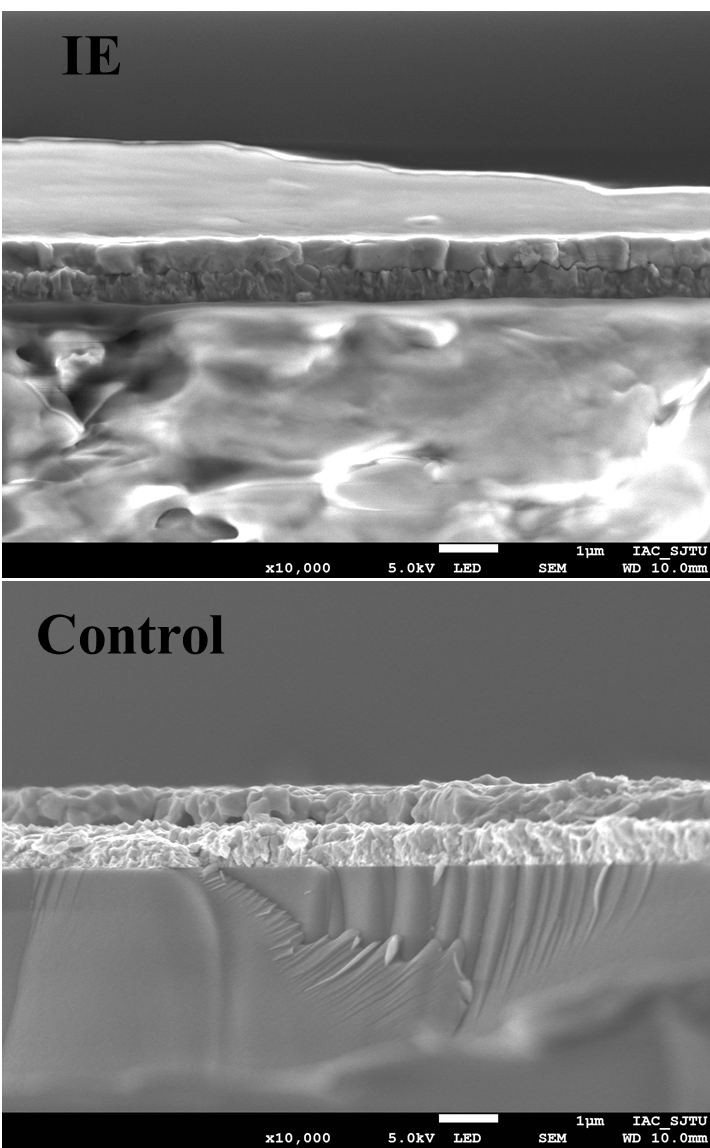


**Figure S7.** Cross-section SEM images of the perovskite films prepared from IE and control precursor solutions.

**Figure S8.** UV-vis spectrums of IE perovskite films before and after aged on a 100°C hotplate for 10 days in N_2_ glovebox.


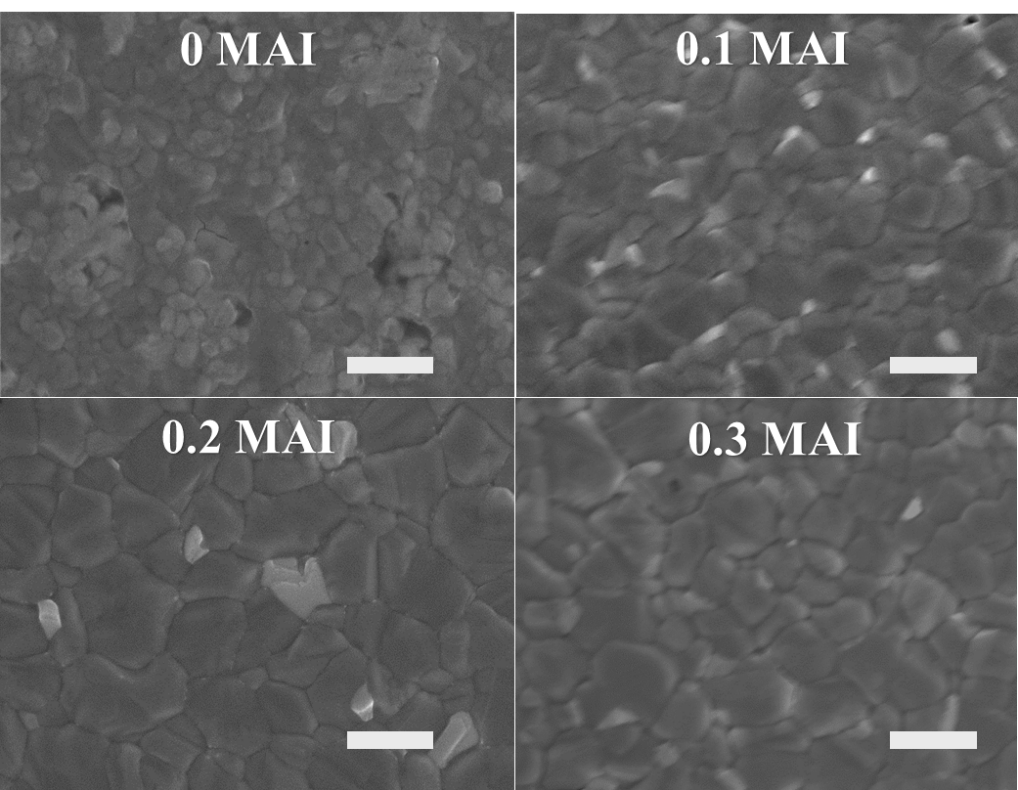


**Figure S9.** Comparison of SEM images of the annealed perovskites films with different MAI contents, the scalebar is 1µm. The grain size of 0.2MAI is larger than that of 0.1MAI, but grains on the perovskite films of 0.3MAI are no larger than the 0.2MAI with pinholes starting to appear.

**Figure S10.** Comparison of UV-vis spectra of annealed perovskite films with different MAI contents. The absorbance value of 0.3MAI is also weaker than 0.2MAI.

**Figure S11.** *J-V* curves of perovskite solar cell devices fabricated by precursor with different MAI contents. The 0.2MAI recipe shows better performance than others.


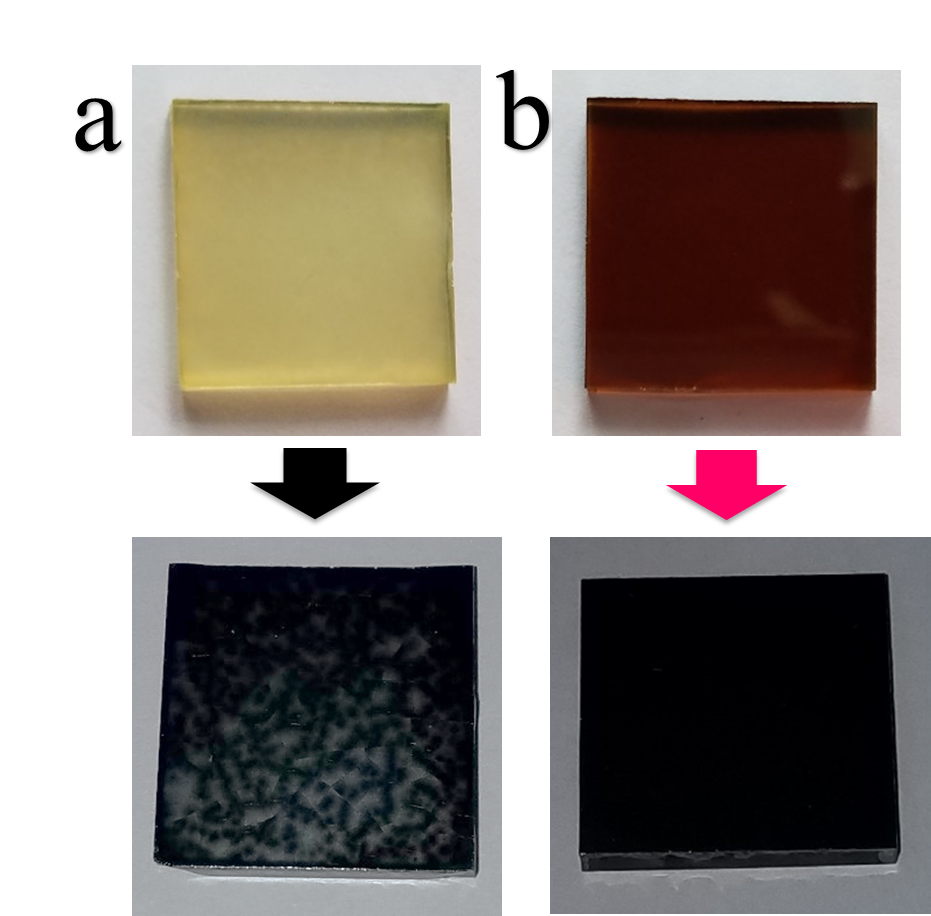


**Figure S12.** Photos of perovskite films using control (a) and IE precursor (b) at initial (top) and final (bottom) annealing stages.

**Figure S13.** Enlarged XRD pattern of Figure 2b, no peaks of MAPbI_3_ could be observed.


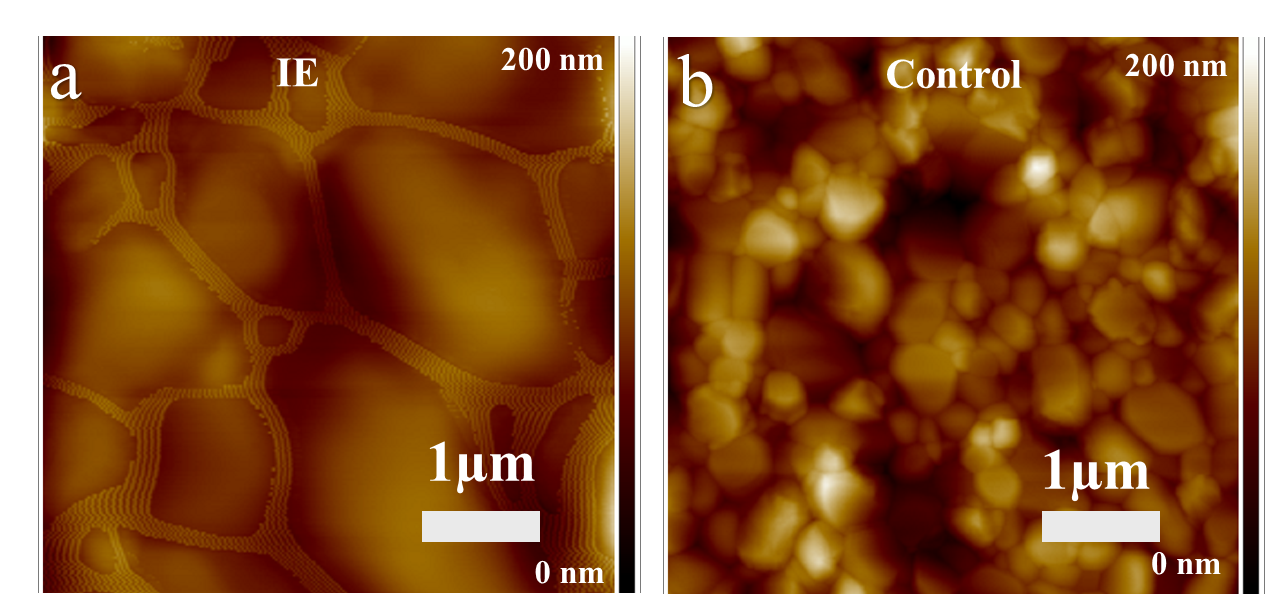


**Figure S14.** AFM images of IE (a) and control (b) precursor films.

**Figure S15.** The PL spectra of the control precursor film collected from the perovskite side and the glass side.

**Figure S16.** The PL spectra of the annealed IE film collected from perovskite side and glass side.

**Figure S17.** The UV-vis spectra revolution of the control films during annealing process.

**Figure S18.** XRD pattern evolution of the control precursor films with different annealing durations.


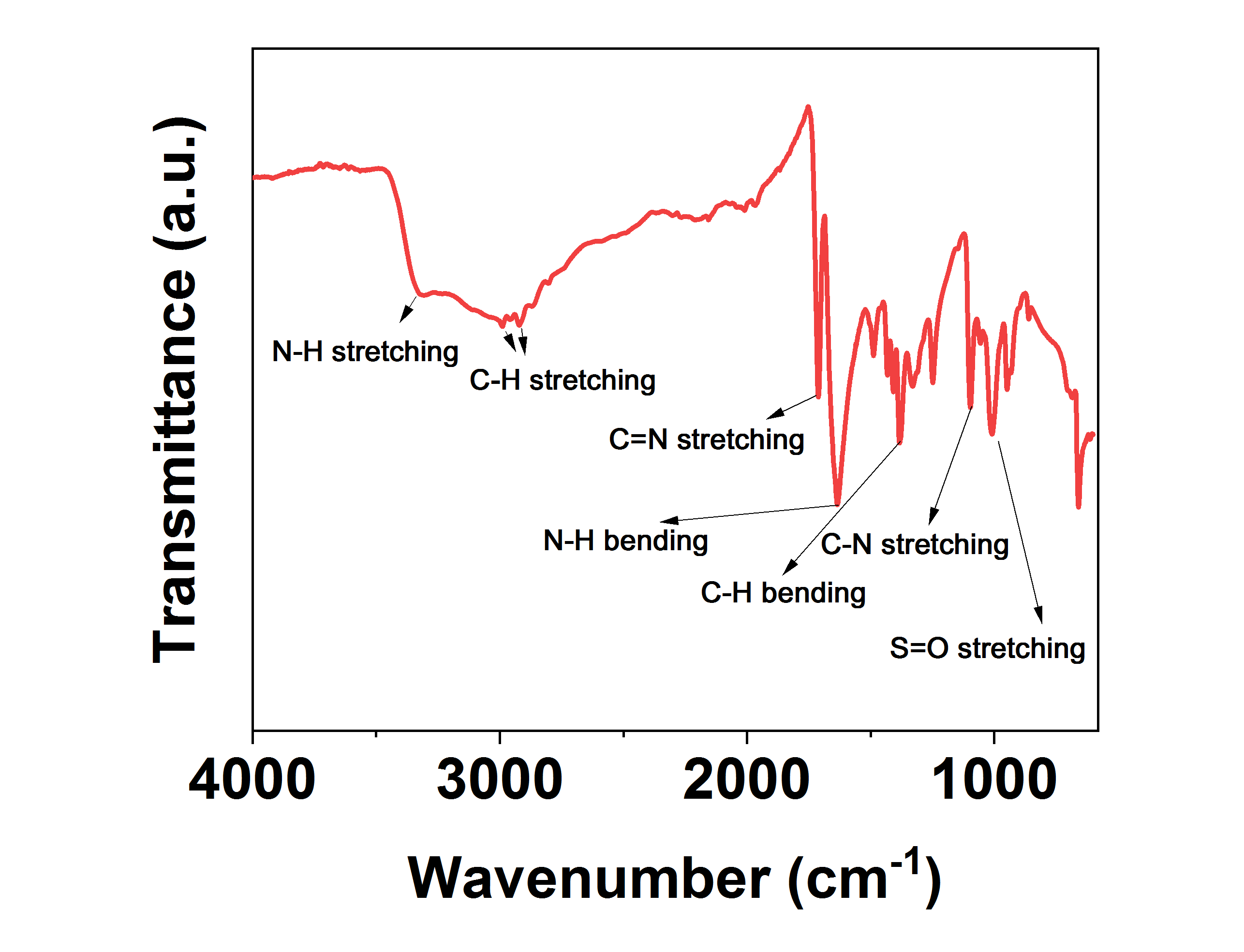


**Figure S19.** Fourier transform infrared spectroscopy (FT-IR) spectra of the IE precursor film after anti-solvent dipping.

**Figure S20.** XRD patterns of the IE precursor films with different annealing procedure. The bottom annealing represents putting the precursor film onto a hotplate right after the anti-solvent dipping while the top annealing was realized by placing the precursor film under a hot air flow right after the anti-solvent dipping.

**Figure S21**. Tof-sims of annealed control and IE samples, no signals of MA^+^ could be observed.

**Table S1.** Summary on device performance parameters of FA_0.95_Cs_0.05_PbI_3_ (both IE and control types) based perovskite solar cells from 32 devices. Upper row: values of the champion device, lower row: the statistical values.

| **Precursor Type** | ***J_sc_* (mA/cm^2^)** | ***V*_oc_ (V)** | **FF** | **PCE (%)** |
| --- | --- | --- | --- | --- |
| Control | 22.61 | 1.03 | 0.721 | 16.79 |
|  | (22.656±0.629) | (1.01±0.034) | (0.701±0.019) | (15.857±0.757) |
| IE | 25.40 | 1.13 | 0.805 | 23.11 |
|  | (24.941±0.253) | (1.088±0.014) | (0.792±0.015) | (21.484±0.499) |
